# Analysis of Epigenetic Alterations in Homologous Recombination DNA Repair Genes in Male Breast Cancer

**DOI:** 10.3390/ijms21082715

**Published:** 2020-04-14

**Authors:** Saudade André, Sandra P. Nunes, Fernanda Silva, Rui Henrique, Ana Félix, Carmen Jerónimo

**Affiliations:** 1 Department of Pathology, Portuguese Oncology Institute of Lisboa, 1099-023 Lisboa, Portugal; ana.felix@nms.unl.pt; 2 Cancer Biology & Epigenetics Group—Research Center, Portuguese Oncology Institute of Porto (CI-IPOP), 4200-072 Porto, Portugal; sandra.pinto.nunes@ipoporto.min-saude.pt (S.P.N.); henrique@ipoporto.min-saude.pt (R.H.); 3 Medical School, NOVA University, 1169-056 Lisbon, Portugal; fernanda.silva@nms.unl.pt; 4 Department of Pathology, Portuguese Oncology Institute of Porto, 4200-072 Porto, Portugal; 5 Department of Pathology and Molecular Immunology, Institute of Biomedical Sciences Abel Salazar– University of Porto (ICBAS-UP), 4050-313 Porto, Portugal

**Keywords:** male breast cancer, epigenetics, homologous recombination DNA repair, detection

## Abstract

Background: Male breast cancer (BC) is a distinct neoplasm with low but rising incidence, frequently diagnosed as advanced stage disease. Considering the relevance of altered homologous recombination repair (HRR) in male BC, we aimed to explore the biomarker potential of aberrant promoter methylation of *ATM*, *BRCA1*, *PALB2*, *RAD51B,* and *XRCC3*. Methods: Formalin-fixed paraffin-embedded (FFPE) tissue samples from 128 male BC patients, paired adjacent normal tissue and 19 gynecomastia cases were collected and assessed by quantitative methylation-specific PCR (qMSP). Non-parametric tests were used to compare methylation levels between tumor and non-tumor samples and to seek for associations with clinicopathological variables. Results: Only *RAD51B* and *XRCC3* disclosed significant differences between tumor and gynecomastia (*p* < 0.0001 and *p* = 0.020, respectively). Assembled in a panel, *RAD51B* and *XRCC3* promoter methylation discriminated male BC from gynecomastia with 91.5% sensitivity, 89.5% specificity, and 91.2% accuracy. Moreover, promoter methylation levels were lower in paired non-tumor tissues, comparing to tumor samples. No associations were found between epigenetic alterations and clinicopathological features, as well as with RAD51 and XRCC3 immunoexpression and methylation levels. Conclusion: Quantitative promoter methylation of *RAD51B* and *XRCC3* constitutes a promising and accurate biomarker for male BC. Validation in larger series and in liquid biopsies is warranted to confirm its usefulness in detection and monitoring settings.

## 1. Introduction

Male breast carcinoma (BC) is a multifactorial neoplasm lacking specific guidelines for detection, therapy and surveillance. Although it constitutes a rare entity, comparatively to its female counterpart, incidence has been rising over the last decades [1,2]. Furthermore, advanced stage disease is rather common at diagnosis [1]. Although several genetic, hormonal, and environmental risk factors have been acknowledged, an in-depth understanding of the biologic peculiarities of male breast carcinogenesis is clearly lacking [3].

The process of carcinogenesis is complex, resulting from the accumulation of multiple genetic and epigenetic alterations [4]. The best characterized epigenetic change in cancer consists on altered methylation of CpG dinucleotides, impacting on genome stability and regulation of gene expression [5]. Aberrant methylation, occurring mostly at gene promoter regions, is associated with gene transcription repression [5]. This alteration is among the most common and earliest events involved in cancer initiation and promotion, being easily measured [6].

Homologous recombination repair (HRR) is a major surveillance mechanism in the preservation of genome integrity, acting in repair of DNA double-strand breaks, which occur during replication [7]. *BRCA2*, the most common high penetrance susceptibility gene for male BC, but also *ATM, BRCA1, PALB2, RAD51,* and *RAD51* paralogs play important roles in HRR pathway [8]. *RAD51* paralogs encode for proteins that structurally resemble RAD51 and congregate in vivo into three subcomplexes, comprising *BCDX2 (RAD51B, RAD51C, RAD51D, XRCC2), CX3 (RAD51C, XRCC3*), and the Shu complex (*SWSAP1, SWS1*) [7,8,9,10,11,12]. Indeed, the balance between BRCA2, RAD51 and RAD51 paralogs seems to be essential in HRR [8,12,13]. Mutations in HRR genes, either somatic and/ or in the germline occur in multiple conditions, including hereditary breast and ovarian cancer susceptibility syndromes, in which there is also increased male BC risk [2,8,14,15,16,17]. Nonetheless, HRR deficiency may also be mediated by DNA repair gene aberrant promoter methylation. Although altered DNA methylation has been seldom reported in male BC, it might constitute a novel biomarker for disease detection and monitoring, allowing for more personalized clinical care [4,18,19,20,21].

Owing to the relevance of HRR deficiency in male BC and the lack of systematic studies on altered methylation patterns of HRR genes in this specific context, we aimed to explore the epigenetic signature of the HRR genes *ATM, BRCA1, PALB2, RAD51B,* and *XRCC3* in a large, well characterized (clinically and pathologically) series of male BC patients, to identify novel detection, diagnostic and/or prognostic biomarkers that might perfect clinical management.

Additionally, 19 cases of gynecomastia were added as benign comparative model. Gynecomastia is the most common benign disease in male breast and shares risk factors with male BC, including high estrogen levels [18,22]. However, gynecomastia is not considered by itself a risk factor for male BC [22].

## 2. Results

### 2.1. Clinical and Pathological Data

This study included 128 male BC, matched normal tissues (66 normal breast tissue and 62 axillary lymph nodes) and 19 patients with gynecomastia. Detailed clinicopathologic characterization of the male BC cases is provided in Table 1. The mean age of patients with breast cancer at diagnosis was 66.7 years (range: 37–87 years). About 20% of the male BC patients had a familial history (FH) of breast cancer. Germline BRCA1 mutations were not found in this series. Germline BRCA2 mutations were found in 12 patients (32.4%). Ten (83.3%) of these 12 patients had a FH of breast cancer. Six patients (4.7%) had bilateral carcinoma and 20 patients (15.6%) had non-breast primary neoplasm (NBPN), most of them (eight patients—40%) corresponding to prostate cancer. Germline BRCA mutations were evaluated in 12 patients with NBPN and BRCA2 was identified in four (33.3%) of these patients, all with a FH of BC (Table 2).

Patients with gynecomastia were younger, with a mean age of 34.3 years (range: 16–69 years). None of the patients with gynecomastia had FH of breast cancer. Twelve cases were bilateral. Five patients had NBPN, two of which were prostate carcinomas. One patient with gynecomastia and prostate carcinoma harbored a germline *BRCA2* mutation.

### 2.2. Gene Promoter Methylation Levels

**Figure 1:**
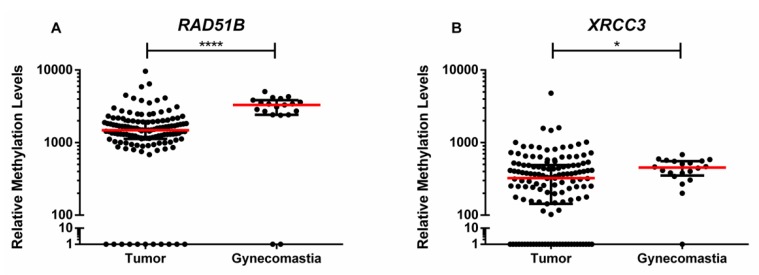
Scatter plot of the distribution of (**A**) *RAD51B* and (**B**) *XRCC3* relative methylation levels [(gene/β-Actin) × 1000] of tumor tissue samples (*n* = 128) and gynecomastia tissue samples (*n* = 19). Red horizontal line represents the median levels and the black lines the interquartile range. *p* value obtained by Mann–Whitney U test, * *p* < 0.05 and **** *p* < 0.0001.

*ATM*, *BRCA1*, *PALB2*, *RAD51B,* and *XRCC3* promoter methylation levels were evaluated in paired tumor, normal tissue, and adjacent lymph nodes, and in gynecomastia tissue samples. Only *RAD51B* and *XRCC3* disclosed statistically significant differences between tumor and gynecomastia tissues, with higher methylation levels observed in gynecomastia tissue samples (Table 3, Figure 1).

**Figure 2:**
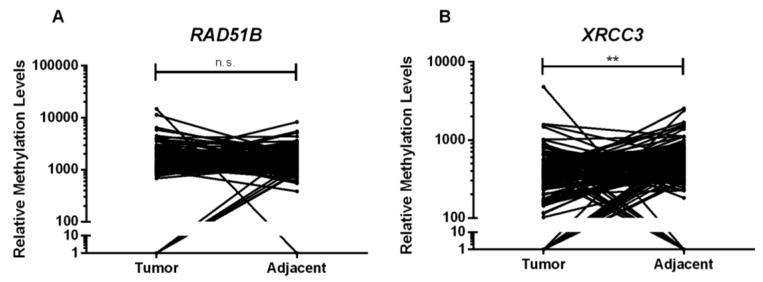
Relative methylation levels distribution of (**A**) *RAD51B* and (**B**) *XRCC3* of tumor tissue samples (*n* = 128) and normal adjacent tissue samples (*n* = 128). *p* value obtained by Wilcoxon signed-rank test, n.s. *p* > 0.05 and ** *p* < 0.01.

Furthermore, *XRCC3* promoter methylation levels were lower in normal adjacent tissue comparing to tumor tissue (*p* = 0.002), whereas *RAD51B* promoter methylation levels were higher in tumor samples, although not reaching statistical significance (*p* = 0.968) (Figure 2). No differences were depicted for the remainder genes.

### 2.3. Biomarker Performance

The gene promoters that showed statistically significant differences between tumor and gynecomastia samples (*RAD51B* and *XRCC3*) were evaluated as potential biomarkers for male BC. Individually, *RAD51B* displayed over 80% sensitivity and specificity, whereas *XRCC3* correctly identified 43.4% of the tumor samples with 94.7% specificity (Table 4).

**Figure 3:**
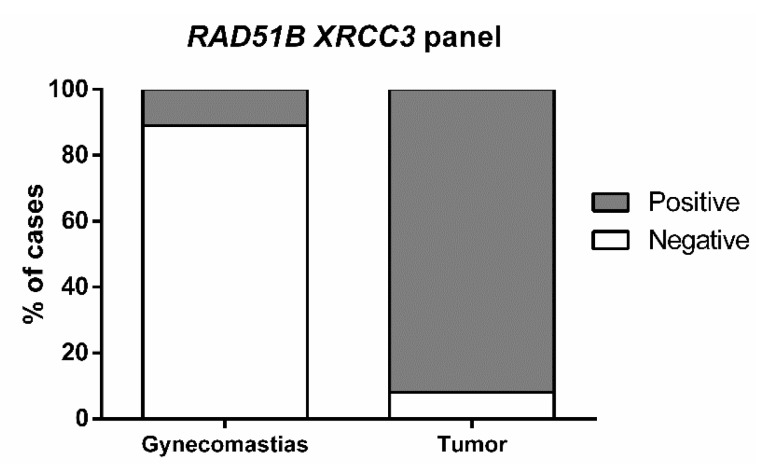
Percentage of cases detected by *RAD51B XRCC3* gene panel in tumor tissue samples (Positive 92%, Negative 8%) and in gynecomastia tissue samples (Positive 89%, Negative 11%).

When the two genes were assembled in a panel, sensitivity increased to 91.5%, with 89.5% specificity and 91.2% accuracy for identification of male BC vs. gynecomastia (Table 5, Figure 3).

No statistically significant associations between epigenetic alterations in the tested HRR genes and clinicopathological parameters were depicted, either in male BC or gynecomastia cases.

### 2.4. Immunohistochemistry

**Figure 4:**
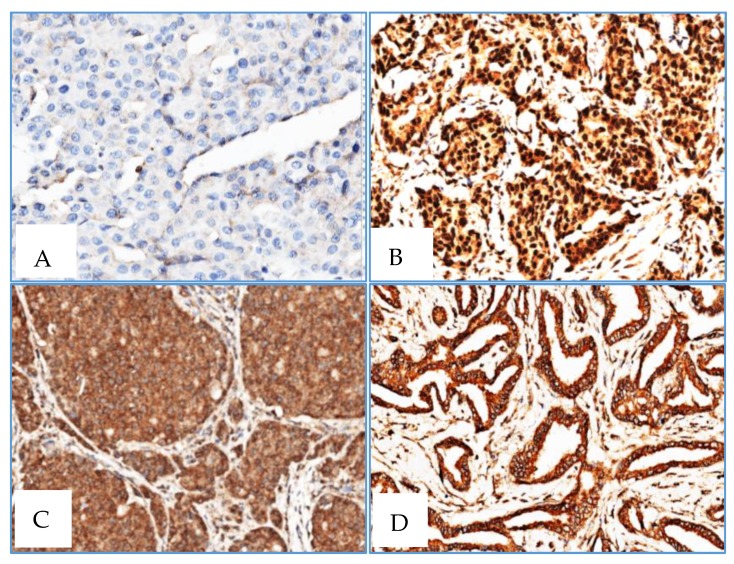
Male BC: XRCC3 (**A**) negative staining with XRCC3 methylation level = 0 in male BC (1 case); XRCC3 (**B**) positive nuclear and cytoplasmatic staining in male BC (1 case); RAD51 (**C**) and XRCC3 (**D**) negative nuclear staining and positive cytoplasmatic staining in male BC (33 and 32 cases) (×400).

Almost all cases depicted positive cytoplasmatic staining, whereas RAD51 and XRCC3 nuclear staining was only observed in one case (Table 6). RAD51 and XRCC3 nuclear and cytoplasmatic staining was also observed in one male BC patient No associations were found between RAD51 and XRCC3 immunoexpression and methylation levels (Figure 4).

**Figure 5:**
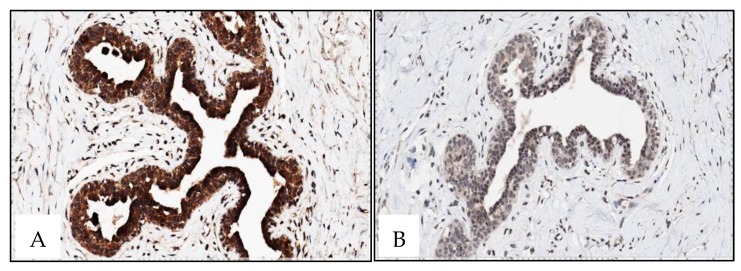
Gynecomastia: RAD51 (**A**) positive intense nuclear and cytoplasmatic staining; XRCC3 (**B**) negative nuclear staining and weak cytoplasmatic staining (×400)**.**

RAD51 and XRCC3 exhibited consistent staining patterns both in gynecomastia and female breast normal tissue: Intense positive nuclear and cytoplasmatic staining was found for RAD51 in all samples, whereas negative nuclear staining and a weak cytoplasmatic staining was observed for XRCC3 (Figure 5). Nonetheless, no inverse correlation was found between methylation and imunoexpression status.

## 3. Discussion

Male BC is a multifactorial and distinctive neoplasia with low, but rising, incidence, requiring a personalized approach and warranting optimal care [1,23]. To achieve this aim, detailed knowledge of genetic and epigenetic alterations, as well as of other specific characteristics of BC in the male gender are mandatory [23]. Population-based mammographic screening in males has no role considering the rarity of male BC, although it may be useful in selected high-risk groups [1,24]. In current clinical practice, male BC is diagnosed by mammography or ultrasonography and confirmed by core biopsy, which is always performed following a suspicious clinical examination and frequently at advanced disease stage [1]. Furthermore, specific biomarkers that might assist in early disease detection, diagnosis, and prognostication are clearly lacking and constitute an unmet need. We, thus, assessed the methylation status of five genes (*ATM, BRCA1, PALB2, RAD51B,* and *XRCC3*) involved in HRR (which is deficient in a large proportion of male BC cases) looking for biomarkers that might be useful for clinical management.

Notwithstanding the biological peculiarities of male BC, the uselessness of mammographic screening due to low incidence rates, the high incidence of gynecomastia that may have overlapping clinical presentation, the particular anatomic characteristics of the male breast, the absence of publicly-available information about the disease, the prevalence in old age groups and the fact that males are less likely to report symptoms that would guide to an early diagnosis, contribute to the significant number of advanced stage disease at diagnosis and, consequently, the high mortality rates of male BC [25]. Indeed, in this cohort, 39.1% of the patients presented at stages III and IV and 31.3% died of disease, underlining the importance of early diagnosis. Furthermore, evidence of significant hereditary predisposition was found in this cohort (FH of breast cancer, *BRCA2* mutations and NBPN, in 20.3%, 32.4%, and 15.5% of cases, respectively) which is line with published literature [26,27]. Furthermore, the clinical and pathological characteristic of the patient population are also similar to those previously published [3,21,28], which further validates our dataset. 

Although the acquisition of epigenetic alterations, in general, and aberrant DNA methylation, in particular, is widely acknowledged as an early and relevant event in tumorigenesis [29], these have been seldom reported and with different purposes, in male BC [4,18,19,20,21]. Kornegoor et al., examined promoter methylation of 25 genes in 108 male BCs using methylation specific multiplex ligation dependent probe amplification and concluded that promoter methylation was common in male BC and that high methylation status correlated with aggressive phenotype and poor outcome [18]. Subsequently, Pinto et al. found different expression patterns in male and female familial BC in a set of 27 familial BC cases, using qMSP [19]. Johanssen et al. performed a genome-wide methylation profiling of 47 male BC, underscoring the heterogeneity of this entity and suggesting that male BC should not be defined using conventional criteria applied to female breast cancer [20]. Using methylation-sensitive high resolution, Deb et al. assessed a panel of 10 genes in 60 male BCs, concluding that *BRCA2*-associated male BC was characterized by high gene methylation and that the average methylation index might be a useful prognostic marker [4]. Finally, Rizzolo et al. assessed promoter methylation in 69 male BC patients and concluded that alterations in methylation patterns were common in BC and might identify specific subgroups related to *BRCA1/2* mutation status and some clinicopathologic parameters [21].

Among the five gene promoters tested, only two *- RAD51B* and *XRCC3*—disclosed statistically significant differences between tumor and gynecomastia tissue samples, whereas *ATM, BRCA1,* and *PALB2* did not. Nevertheless, because only 19 gynecomastia tissues were included in this study, the results must be interpreted with caution and further validation using an independent cohort is required. Furthermore, *RAD51B* and *XRCC3* promoter levels were higher in tumor tissues compared to normal breast or lymph node, although with statistical significance for *XRCC3*, only. Globally, these results are in line with those of Kornegoor et al., which found that *ATM* and *BRCA1* promoter methylation did not seem to play a key role in male BC genesis [18]. Interestingly, *RAD51B* and *XRCC3* promoter methylation has been reported in association with the inflamed phenotype of squamous cell carcinomas of the head and neck, lung, and cervix, warranting further investigation as predictive biomarkers of response to immunotherapy [30].

Gynecomastia is a common benign proliferation of the breast that shares with male BC the risk factors related to high estrogen levels and its discrimination from BC is clinically challenging, being not considered a risk factor for male BC [22]. Thus, it was selected as control for determining the biomarker performance of gene promoter methylation as this constitutes a clinical scenario in which specific biomarkers might aid in differential diagnosis and monitoring. Remarkably, the methylation panel combining *RAD51B* and *XRCC3* accurately discriminated male BC from gynecomastia, in tissue samples. This might prove useful in the diagnostic context of biopsies with limited tissue representativeness but translation into monitoring scenarios requires the validation of this performance in liquid biopsies. Nevertheless, it should be emphasized that this gene methylation panel constitutes the first discriminative biomarker in this setting.

Interestingly, normal breast tissues disclosed *RAD51B* and *XRCC3* promoter methylation, although with lower median promoter methylation levels compared to BC, suggesting the existence of a cancerization field effect. This phenomenon reflects the susceptibility of normal tissue to undergo early genetic and epigenetic alterations leading to tumor development [31]. Field cancerization was hypothesized to explain the development of multifocal areas of premalignant change, multiple primary tumors and local recurrence [32] and more recent studies demonstrate that aberrant DNA methylation patterns, either hyper- or hypomethylation, are potential biomarkers of field cancerization and may be useful for risk stratification [33]. Surprisingly, however, higher *RAD51B* and *XRCC3* promoter methylation levels were disclosed in gynecomastia comparing to male BC. This finding might be related with proliferation, high estrogen levels or other yet unknown risk factors. To understand the biological consequences of these observations, expression analysis of those genes was performed, but no significant associations were disclosed between methylation levels and RAD51 and XRCC3 expression for gynecomastia and BC lesions, as well as for adjacent normal breast tissue. However, the only antibody available is not specific for RAD51B protein. Moreover, promoter methylation acts in concert with other epigenetic mechanisms (e.g., histone post-translational modifications and chromatin remodeling) to achieve effective gene silencing. Thus, although RAD51B and XRCC3 promoter methylation levels might be higher in gynecomastia, histone-related factors might preclude effective gene silencing, contrarily to BC. Notwithstanding the elusive biological significance of this finding, RAD51B and XRCC3 promoter methylation stand as candidate biomarkers for male BC, requiring further exploitation, namely in liquid biopsies by analyzing circulating cell-free DNA methylation in plasma or serum samples, which could better represent tumor heterogeneity and allow for a minimally-invasive strategy for BC detection.

## 4. Materials and Methods

### 4.1. Patients and Samples Collection

A cohort of 128 male BC patients, diagnosed and treated at the Portuguese Oncology Institute of Lisbon (Lisbon, Portugal), between 1978 and 2018 were enrolled, after informed consent. Routine sampling for standard pathological examination by H&E and immunostaining was performed, allowing for tumor classification, grading and staging [34]. A representative formalin-fixed, paraffin-embedded (FFPE) tumor tissue sample was made available for molecular analyses. The corresponding adjacent normal breast tissue and lymph nodes were also included in the study, as controls. For comparison purposes, 19 cases of gynecomastia were used. Patient data, including age, family history, tumor bilaterality, presence of non-breast primary neoplasms, information about distant metastasis and follow-up were obtained from clinical records. Germline mutational *BRCA2* status was evaluated in 37 cases of male BC and in one case of gynecomastia, as previously described [25]. This study was approved by the Ethics Committee of Portuguese Oncology Institute of Lisbon (UIC/859 in 12/11/2013).

### 4.2. DNA Extraction and Sodium-Bisulfite Modification

Areas of interest (breast cancer, normal breast and gynecomastia) were delimited in H&E slides by a dedicated Pathologist (S.A.), macrodissected from 10 µm tissue sections, deparaffinized with xylene (VWR, Radnor, PA, USA) and rehydrated using 100% ethanol (Merck Millipore, Burlington, MA, USA). DNA was extracted using the FFPE RNA/DNA Purification Plus Kit (Norgen Biotek, Thorold, ON, Canada) according to the manufacturer’s recommendations. DNA samples were eluted in 20 µL of sterile distilled water and stored at −20 °C until further use. DNA was quantified using the Qubit 4 Fluorometer (Invitrogen, Carlsbad, CA, USA), using the manufacturer’s recommendations.

Sodium-bisulfite modification was performed in all samples using the EZ DNA Methylation-Gold^TM^ (Zymo Research, Orange, CA, USA) following the manufacturer’s recommendations. 150 ng of extracted DNA were used and eluted in 60 µL of sterile distilled water. Additionally, 1000 ng of CpGenome^TM^ Universal Methylated DNA (Millipore, Temecula, CA, USA) was sodium-bisulfite converted for control purposes and eluted in 40 µL of sterile distilled water. All sodium-bisulfite converted DNA was stored at −80 °C until further use.

### 4.3. Quantitative Methylation-Specific PCR (qMSP)

*ATM*, *BRCA1*, *PALB2*, *RAD51B,* and *XRCC3* (Supplementary Figure S1) promoter methylation levels were assessed by qMSP, using *β-Actin* as a reference gene. The reactions were carried out in 384-well plates using the LightCycler 480 Instrument (Roche Diagnostics, Manhaeim, Germany) and the sodium-bisulfite modified DNA was used as a template. The primers’ volumes and conditions used for each gene are listed in Supplementary Table S1. Per well, 2 µL of sodium-modified DNA and 5 µL of Xpert Fast SYBR (GRiSP, Porto, Portugal) were added. All samples were run in triplicate. In order to generate a standard curve for DNA relative quantification and plate efficiency calculation, the sodium-bisulfite modified CpGenome^TM^ Universal Methylated DNA was subjected to serial dilutions (5× dilution factor). Efficiency values above 90% were considered for each plate. Relative methylation levels were obtained by calculating the ratio between the methylation levels of each gene and the respective value of *β-Actin*, multiplying by 1000 for easier tabulation.

### 4.4. Immunohistochemistry

Immunohistochemical staining was performed on 4-μm-thick, formalin-fixed, paraffin-embedded tissue sections from 3 tissue microarrays (TMAs) that included 33 male BC cases and 10 gynecomastia cases and one TMA including 22 normal female breast tissue (cases retrieved from patients with age range from 40 to 70 years-old). Slides were stained on the BenchMark ULTRA IHC/ISH Automatic staining platform (Ventana Medical Systems) with anti-human XRCC3 (dilution 1:20, for 40 min; pretreatment ULTRA CC1-72 min, catalogue number SAB4503092, Sigma), anti-human RAD51 antibody (dilution 1:700 for 40 min; pretreatment ULTRA CC1-48 min, catalog number SAB1406364, Sigma), with appropriate positive and negative controls samples. Antigen detection was performed using OptiView DAB IHC Detection Kit (Ventana Medical Systems) with diaminobenzidine as the chromogen to detect antigen expression. Tissue sections were counterstained with Mayer's hematoxylin.

### 4.5. Statistical Analysis

Non-parametric tests were used to compare the methylation levels between tumor and non-tumorous samples and to assess associations with clinicopathological variables (Kruskall–Wallis test for three or more groups, followed by pairwise comparisons using Mann–Whitney U test with Bonferroni’s correction, when applicable, and Wilcoxon signed-rank test for paired samples). Correlations between age and genes’ methylation levels were evaluated by Spearman’s nonparametric test. Receiver operating characteristic (ROC) curve analysis was performed and the validity estimates (sensitivity, specificity, and accuracy) were calculated to assess biomarker performance. Tumor samples of patients subjected to neoadjuvant therapy were not considered for this analysis. Samples were categorized as methylated (positive) or unmethylated (negative) based on the cut-off determined by ROC curve analysis corresponding to the highest sensitivity and specificity (Youden’s J index) [35].

## 5. Conclusions

In this study we demonstrated that promoter methylation levels of *RAD51B* and *XRCC3* differ between male BC and gynecomastia tissues, suggesting its usefulness, in a panel, as male BC biomarkers. Further analyses in liquid biopsies are mandatory to assess the potential of this panel for early detection, in at-risk populations, and disease monitoring.

## Figures and Tables

**Table 1:** Clinicopathological characteristics of male breast cancer patients.

Characteristics	Number	Number (%)
Age (years)	128	
37–69		67 (52.3%)
≥70		61 (47.7%)
Familial history (FH) of breast cancer	128	
No		102 (79.7%)
Yes		26 (20.3%)
Bilateral breast cancer	128	
No		122 (95.3%)
Yes		6 (4.7%)
Non-breast primary neoplasms (NBPN)	128	
No		108 (84.4%)
Yes		20 (15.6%)
Tumor size (pT)	128	
pTis		8 (6.2%)
pT1		31 (24.2%)
pT2		43 (33.6%)
pT3		2 (1.6%)
pT4		44 (34.4%)
Axillary nodal status (pN)	128	
pN0		60 (46.9%)
pN1		68 (53.1%)
Distant metastasis (M)	128	
M0		122 (95.3%)
M1		6 (4.7%)
Anatomic stage (AS)	128	
0		8 (6.2%)
I		23 (20%)
II		47 (36.7%)
III		43 (33.6%)
IV		7 (5.5%)
Histological type (HT)	120 ^1^	
Invasive no special type (NST)		112 (93.3%)
Other invasive subtypes		8 (6.7%)
Histological grade (G)	120 ^1^	
G1		20 (16.7%)
G2		74 (61.7%)
G3		26 (21.6%)

^1^ Excluding eight in situ carcinomas.

**Table 2:** Molecular characteristics and follow-up of male breast cancer patients.

Characteristics	Number	Number (%)
Germline BRCA2 mutations	37	
Indeterminate		25 (67.6%)
Positive		12 (32.4%)
Estrogen receptor (ERα) status	128	
Positive		125 (97.7%)
Negative		3 (2.3%)
Progesterone receptor (PR) status	120 ^1^	
Positive		97 (81%)
Negative		23 (19%)
ERBB2 (IHC and ISH) status	120 ^1^	
Negative		111 (92.5%)
Positive		9 (7.5%)
Ki67 immunoreactivity	120 ^1^	
Low		77 (64.2%)
High		43 (35.8%)
Clinically defined subtypes	120 ^1^	
Luminal A-like		44 (36.7%)
Luminal B-like		64 (53.3%)
HER2-like		9 (7.5%)
Triple negative		3 (2.5%)
Follow-up	128	
Died of disease		40 (31.3%)

^1^ Excluding eight in situ carcinomas.

**Table 3:** Genes’ methylation levels *p* values comparing tumor and gynecomastia tissues.

Gene	*p* Value
*ATM*	0.749
*BRCA1*	0.289
*PALB2*	0.436
*RAD51B*	<0.0001
*XRCC3*	0.020

**Table 4:** Biomarker performance detection of *RAD51B* and *XRCC3* hypomethylation levels in tissue samples.

Validity Estimates	*RAD51B*	*XRCC3*
Sensitivity %	82.9	43.4
Specificity %	94.7	94.7
Accuracy %	84.7	51.2

**Table 5:** Biomarker performance detection of *RAD51B* and *XRCC3* hypomethylation gene panel levels in tissue samples.

Validity Estimates	*RAD51B XRCC3*
Sensitivity %	91.5
Specificity %	89.5
Accuracy %	91.2

**Table 6:** AD51 and XRCC3 immunohistochemical expression in Male BC

Immunoexpression	RAD51 (Number of Cases)	XRCC3 (Number of Cases)
Positive cytoplasmatic	33 (all)	32
Positive nuclear and cytoplasmatic	1	1
Negative nuclear and cytoplasmatic	0	1

## Supplementary Materials

The following are available online at https://www.mdpi.com/1422-0067/21/8/2715/s1. Supplementary Table S1: Primers sequences, annealing temperature and final concentration in the PCR reaction. Supplementary Figure S1: Gene location of the CpG islands and amplicons for ATM, BRCA1, PALB2, RAD51B and XRCC3. Blue arrow represents the gene promoter; Yellow boxes represent CpG islands; Red arrows represent the primers and amplicon; Numbers below the yellow boxes and next to the red arrows stand for the gene coordinates based on Human GRCh38/hg38 Assembly.

## Abbreviations

BC	Breast cancer
FFPE	Formalin-fixed paraffin-embedded
FH	Familial history
HRR	Homologous recombination repair
NBPN	Non-breast primary neoplasm
ROC	Receiver operating characteristic
qMSP	Quantitative methylation-specific PCR
