# Supplementary material for: Analysis of Epigenetic Alterations in Homologous Recombination DNA Repair Genes in Male Breast Cancer
Source: Int J Mol Sci. 2020 Apr 14;21(8):2715. doi: 10.3390/ijms21082715 (PMC7215617; doi:10.3390/ijms21082715)

Supplementary material
André et al.

Table S1: Primers sequences, annealing temperature and final concentration in the PCR reaction.

Sequences		Anneling temperature	Primers concentration (μM)	Reference
ACTβ	F – 5′ TGG TGA TGG AGG AGG TTT AGT AAG T 3′	60	400	[1]
	R – 5′ ACC AAT AAA ACC TAC TCC TCC CTT AA 3′			
ATM	F – 5′ GGA GTT CGA GTC GAA GGG C 3′	62	400	[2]
	R – 5′ CTA CCT ACT CCC GCT TCC GA 3′			
BRCA1	F – 5′ TTT CGT GGT AAC GGA AAA GC 3′	60	300	[1]
	R – 5′ ATC TCA ACG AAC TCA CGC CG 3′			
PALB2	F – 5′ GTT TCG GTG TCG GTA GGT 3′	62	400	[3]
	R – 5′ ACG AAA TCA AAA TCC TAC G 3′			
RAD51B	F – 5′ AAG ATT TTT AGG GTC GAG AGC 3′	62	400	[4]
	R – 5′ ACG AAA TTT CAC CGT ATT AAC C 3′			
XRCC3	F – 5′ GTT TTC GAG GTT TTG TGG TC 3′	60	400	[4]
	R – 5′ AAC ACG TAT TAC AAA TTC GTT TAA 3′			

References

1. Salta, S.; S, P.N.; Fontes-Sousa, M.; Lopes, P.; Freitas, M.; Caldas, M.; Antunes, L.; Castro, F.; Antunes, P.; Palma de Sousa, S., et al. A DNA Methylation-Based Test for Breast Cancer Detection in Circulating Cell-Free DNA. *J Clin Med* **2018**, *7*, doi:10.3390/jcm7110420.
2. Begam, N.; Jamil, K.; Raju, S.G. Promoter Hypermethylation of the ATM Gene as a Novel Biomarker for Breast Cancer. *Asian Pac J Cancer Prev* **2017**, *18*, 3003-3009, doi:10.22034/apjcp.2017.18.11.3003.
3. Potapova, A.; Hoffman, A.M.; Godwin, A.K.; Al-Saleem, T.; Cairns, P. Promoter hypermethylation of the PALB2 susceptibility gene in inherited and sporadic breast and ovarian cancer. *Cancer Res* **2008**, *68*, 998-1002, doi:10.1158/0008-5472.Can-07-2418.
4. Rieke, D.T.; Ochsenreither, S.; Klinghammer, K.; Seiwert, T.Y.; Klauschen, F.; Tinhofer, I.; Keilholz, U. Methylation of RAD51B, XRCC3 and other homologous recombination genes is associated with expression of immune checkpoints and an inflammatory signature in squamous cell carcinoma of the head and neck, lung and cervix. *Oncotarget* **2016**, *7*, 75379-75393, doi:10.18632/oncotarget.12211.

Supplementary figure 1 – Gene location of the CpG islands and amplicons for *ATM*, *BRCA1*, *PALB2*, *RAD51B* and *XRCC3*. Blue arrow represents the gene promoter; Yellow boxes represent CpG islands; Red arrows represent the primers and amplicon; Numbers below the yellow boxes and next to the red arrows stand for the gene coordinates based on Human GRCh38/hg38 Assembly.

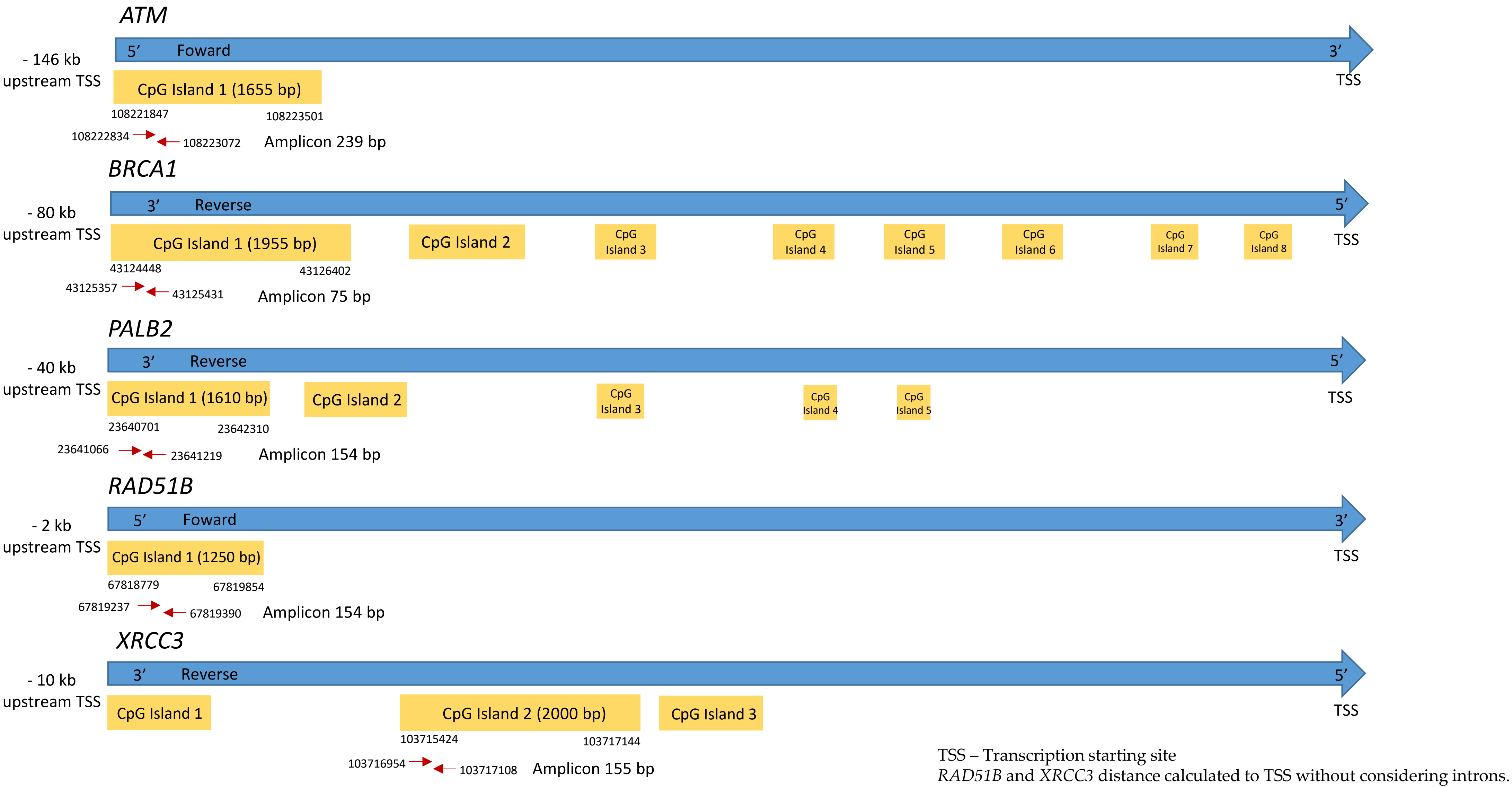

Supplement: Supplementary file 1 [file ijms-21-02715-s001.pdf]
